# An NAD-Specific 6-Hydroxy-3-Succinoyl-Semialdehyde-Pyridine Dehydrogenase from Nicotine-Degrading Agrobacterium tumefaciens Strain S33

**DOI:** 10.1128/spectrum.00924-21

**Published:** 2021-08-11

**Authors:** Jinmeng Shang, Xia Wang, Meng Zhang, Lexin Li, Rufei Wang, Haiyan Huang, Shuning Wang

**Affiliations:** a State Key Laboratory of Microbial Technology, Microbial Technology Institute, Shandong University, Qingdao, People’s Republic of China; b School of Basic Medicine, Shandong First Medical University & Shandong Academy of Medical Science, Jinan, People’s Republic of China; University of Minnesota

**Keywords:** nicotine degradation, aldehyde dehydrogenase, *Agrobacterium tumefaciens*, bio-based furan compounds, furfural, biotransformation

## Abstract

Agrobacterium tumefaciens strain S33 can catabolize nicotine via a hybrid of the pyridine and pyrrolidine pathways. Most of the enzymes involved in this biochemical pathway have been identified and characterized, except for the one catalyzing the oxidation of 6-hydroxy-3-succinoyl-semialdehyde-pyridine to 6-hydroxy-3-succinoylpyridine. Based on a previous genomic and transcriptomic analysis, an open reading frame (ORF) annotated to encode aldehyde dehydrogenase (Ald) in the nicotine-degrading cluster was predicted to be responsible for this step. In this study, we heterologously expressed the enzyme and identified its function by biochemical assay and mass spectrum analysis. It was found that Ald catalyzes the NAD-specific dehydrogenation of 6-hydroxy-3-succinoyl-semialdehyde-pyridine to 6-hydroxy-3-succinoylpyridine. With the nonhydroxylated analog 3-succinoyl-semialdehyde-pyridine (SAP) as a substrate, Ald had a specific activity of 10.05 U/mg at pH 9.0 and apparent *K_m_* values of around 58.68 μM and 0.41 mM for SAP and NAD^+^, respectively. Induction at low temperature and purification and storage in low-salt buffers were helpful to prevent its aggregation and precipitation. Disruption of the *ald* gene caused a lower growth rate and biomass of strain S33 on nicotine but not on 6-hydroxy-3-succinoylpyridine. Ald has a broad range of substrates, including benzaldehyde, furfural, and acetaldehyde. Recombinant Escherichia coli cells harboring the *ald* gene can efficiently convert furfural to 2-furoic acid at a specific rate of 0.032 mmol min^−1^ g dry cells^−1^, extending the application of Ald in the catalysis of bio-based furan compounds. These findings provide new insights into the biochemical mechanism of the nicotine-degrading hybrid pathway and the possible application of Ald in industrial biocatalysis.

**IMPORTANCE** Nicotine is one of the major toxic *N*-heterocyclic aromatic alkaloids produced in tobacco plants. Manufacturing tobacco and smoking may lead to some environmental and public health problems. Microorganisms can degrade nicotine by various biochemical pathways, but the biochemical mechanism for nicotine degradation has not been fully elucidated. In this study, we identified an aldehyde dehydrogenase responsible for the oxidation of 6-hydroxy-3-succinoyl-semialdehyde-pyridine to 6-hydroxy-3-succinoylpyridine; this was the only uncharacterized enzyme in the hybrid of the pyridine and pyrrolidine pathways in Agrobacterium tumefaciens S33. Similar to the known aldehyde dehydrogenase, the NAD-specific homodimeric enzyme presents a broad substrate range with high activity in alkaline and low-salt-containing buffers. It can catalyze not only the aldehyde from nicotine degradation but also those of benzaldehyde, furfural, and acetaldehyde. It was found that recombinant Escherichia coli cells harboring the *ald* gene could efficiently convert furfural to valuable 2-furoic acid, demonstrating its potential application for enzymatic catalysis.

## INTRODUCTION

Nicotine is the main alkaloid produced in tobacco. It is also a toxic *N*-heterocyclic aromatic compound with a high content in tobacco wastes (1). It has been detected in soil, groundwater, and even bottled water (2, 3). Large accumulations of nicotine can cause chromosomal mutations, induce programmed cell death, inhibit cell proliferation, and greatly increase the risk of cardiovascular disease (4, 5). Therefore, the degradation and detoxification of nicotine have become some of the key issues in environmental protection and human health. The traditional chemical method to treat nicotine-containing wastes can cause secondary environmental pollution. In contrast, the microbial degradation of nicotine is highly efficient, harmless, and sustainable (6, 7). A number of microorganisms were found to grow on nicotine, and their degrading pathways were investigated (8). There are two main biochemical pathways for nicotine degradation found in bacteria, a typical pyridine pathway in *Arthrobacter* (9) and a typical pyrrolidine pathway in Pseudomonas (7, 10, 11). Their molecular and biochemical mechanisms for nicotine degradation have been well studied.

**FIG 1:**
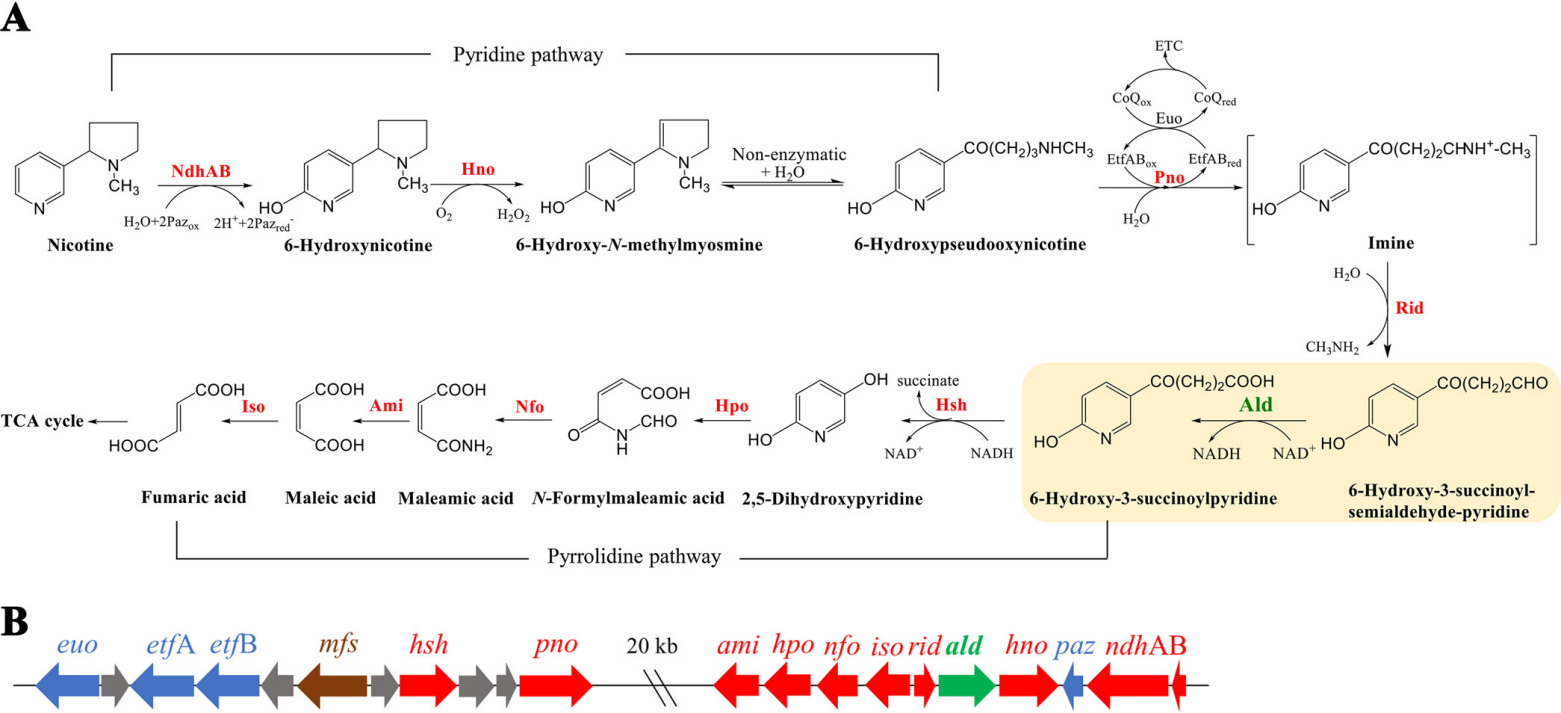
Hybrid pathway of nicotine oxidative degradation by A. tumefaciens S33 (A) and the arrangement of the *ald* gene in the genome of A. tumefaciens S33 (B). NdhAB, nicotine dehydrogenase; Paz, pseudoazurin; Hno, 6-hydroxynicotine oxidase; Pno, 6-hydroxypseudooxynicotine dehydrogenase; EtfAB, electron transfer flavoprotein; Euo, electron transfer flavoprotein:ubiquinone oxidoreductase; ETC, electron transport chain; Rid, reactive imine deaminase; Ald, 6-hydroxy-3-succinoyl-semialdehyde-pyridine dehydrogenase, characterized in this study; Hsh, 6-hydroxy-3-succinoylpyridine hydroxylase; Hpo, 2,5-dihydroxypyridine dioxygenase; Nfo, *N*-formylmaleamate deformylase; Ami, maleamate amidase; Iso, maleate cis-trans isomerase. *mfs*, predicted major facilitator superfamily transporter gene. Green arrow, gene encoding the key enzyme identified in this study; red arrows, genes encoding the characterized key enzymes; blue arrow, genes encoding the enzyme for electron transport; brown arrows, predicted transporter genes; gray arrows, genes with an unknown function.

Recently, we found a hybrid of the pyridine and pyrrolidine pathways, also known as the VPP pathway, for nicotine degradation in Agrobacterium tumefaciens strain S33 (12). This hybrid pathway was also found in *Shinella* sp. strain HZN7 (13) and *Ochrobactrum* sp. strain SJY1 (14). In this pathway (Fig. 1A), nicotine is first converted into 6-hydroxypseudooxynicotine through 6-hydroxynicotine and 6-hydroxy-*N*-methylmyosmine via the pyridine pathway; then, 6-hydroxypseudooxynicotine is converted to 6-hydroxy-3-succinoylpyridine (HSP) through 6-hydroxy-3-succinoyl-semialdehyde-pyridine. Next, the hybrid pathway follows the pyrrolidine pathway through HSP and 2,5-dihydroxypyridine, and finally, it enters the tricarboxylic acid (TCA) cycle. To clarify the biochemical mechanism of this special hybrid pathway, great efforts have been put into studies in recent years, including the analysis of its genome and transcriptome and the purification and characterization of its key enzymes (6, 12, 15–21). Now, a better understanding of the hybrid pathway has been achieved (22) after the key oxidoreductases involved in the pathway, including nicotine dehydrogenase (NdhAB), 6-hydroxynicotine oxidase (Hno), 6-hydroxypseudooxynicotine dehydrogenase (Pno), reactive imine deaminase (Rid), and 6-hydroxy-3-succinoylpyridine hydroxylase (Hsh), were purified and characterized (Fig. 1A) (15–18, 20, 21). However, there are still some unresolved problems; one of which is that the enzyme responsible for converting 6-hydroxy-3-succinoyl-semialdehyde-pyridine to HSP has not yet been identified. Genomic analysis revealed that an open reading frame (ORF) upstream from the *hno* gene was annotated to encode an aldehyde dehydrogenase (Ald) in A. tumefaciens S33 (Fig. 1B) (19). The enzyme from strain S33, designated Ald in this study, is similar to the 3-succinoyl-semialdehyde-pyridine dehydrogenase (SAPD) of Pseudomonas sp. strain HZN6 (34.7% identity of the protein sequence); SAPD was heterologously expressed in Escherichia coli and was found to use NADP^+^ as its coenzyme to catalyze 3-succinoyl-semialdehyde-pyridine (SAP) to 3-succinoylpyridine when the cell extract of the recombinant cells was determined (11). We predicted that the Ald from strain S33 has a similar function to SAPD, using NAD(P)^+^ as a coenzyme to oxidize 6-hydroxy-3-succinoyl-semialdehyde-pyridine to HSP, which works together with Pno to link the pyridine and pyrrolidine pathways. This was also supported by our previous transcriptomic analysis (19), where a log_2_ ratio in fragments per kilobase per million of 6.9 for the expression level of the *ald* gene was observed in nicotine medium versus glucose-ammonium medium. Thus, the *ald* gene may encode an enzyme involved in the oxidative degradation of nicotine.

Aldehyde dehydrogenases are a group of NAD(P)^+^-dependent enzymes that catalyze the oxidation of active aldehyde substances into their corresponding carboxylic acids; they have been applied in multiple fields. For example, a biosensor based on aldehyde dehydrogenase can be used for pesticide residue detection (23, 24) and rapid quantification of alcohols (25). In the chemical industry, aldehyde dehydrogenases can be applied to produce many valuable compounds (26, 27) and effectively degrade toxic environmental pollutants (28). Bio-based furan aldehydes can be converted by green and efficient enzymatic catalysis into high-value-added biochemical products and be used to further produce plastics, pharmaceuticals, and photovoltaic materials (29, 30). Aldehyde dehydrogenase has been demonstrated to play a key role in the catalytic process. Considering that the predicted substrate of Ald in strain S33, 6-hydroxy-3-succinoyl-semialdehyde-pyridine, belongs to aromatic compounds with a pyridine ring, we assumed that this Ald may catalyze various substrates with similar chemical structures, expanding its application in biocatalysis.

In this study, we investigated the function of Ald in nicotine degradation by A. tumefaciens S33 by biochemical analysis and gene disruption. It was verified that Ald catalyzes 6-hydroxy-3-succinoyl-semialdehyde-pyridine to HSP. In addition, Ald can also use SAP instead of the true substrate *in vitro* and oxidize benzaldehyde, furfural, and acetaldehyde. This led us to test its application in transforming furfural, a furan compound derived from the hydrolysis of lignocellulose, into valuable 2-furoic acid using recombinant E. coli cells harboring the *ald* gene. This study provides new evidence for the biochemical mechanism of the hybrid pathway of oxidative nicotine degradation in A. tumefaciens S33 and demonstrates the potential application of Ald in industrial enzymatic catalysis.

## RESULTS

### Recombinant Ald can catalyze the reaction with 6-hydroxy-3-succinoyl-semialdehyde-pyridine as a substrate.

**FIG 2:**
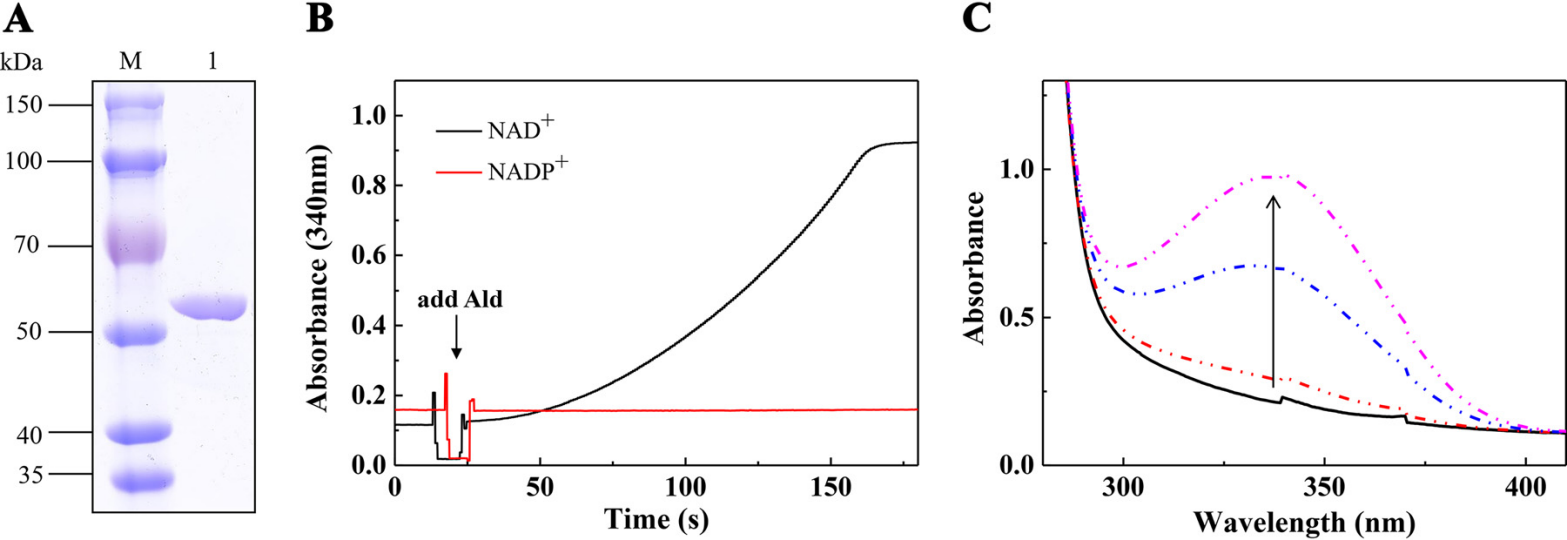
Heterologous expression of Ald and SAP oxidation catalyzed by purified Ald. (A) SDS-PAGE analysis of purified His-tagged recombinant Ald-N. (B) Ald-catalyzed reduction of NAD^+^ (black line) or NADP^+^ (red line) with SAP. The reaction mixture contained 50 mM Gly-NaOH buffer (pH 9.0), 0.1 mM SAP, 1 mM NAD^+^/NADP^+^, and 0.5 μM Ald-N. The reaction was monitored at 340 nm. (C) UV-visible light absorption changes during the Ald-catalyzed NAD-dependent SAP oxidation. The reaction mixture contained 50 mM Gly-NaOH buffer (pH 9.0), 0.1 mM SAP, 1 mM NAD^+^, and 0.5 μM Ald-N.

The *ald* gene of strain S33 was heterologously expressed with the N-terminally fused 6× His tag in E. coli strain BL21 cells. The encoded protein was purified using a HisTrap column and analyzed by SDS-PAGE, which revealed the presence of a band with a molecular mass of about 51 kDa (Fig. 2A), consistent with the calculated mass (50.8 kDa) based on the protein sequence. During the process of purifying Ald, we encountered the problem of protein precipitation. To alleviate its aggregation and precipitation, we reduced the level and speed of protein expression by cultivating the recombinant cells at 16°C overnight, lowered the salt concentration of the buffers during the purification process, and tried various storage buffers. The recombinant protein was found to be relatively stable when purified and stored in 50 mM sodium phosphate buffer (pH 8.0) at 4°C. Gel filtration showed two peaks with molecular masses of 110 kDa and >2,000 kDa, indicating that the purified protein was present in a homodimeric form (Fig. S1, peak b, in the supplemental material) and a multimeric form (Fig. S1, peak a), where the protein could be soluble for a long time.

Then, we tried to measure the activity of Ald. Since the predicted substrate, 6-hydroxy-3-succinoyl-semialdehyde-pyridine, was not commercially available, we prepared it by coupling the reactions catalyzed by Hno and Pno. We first obtained 6-hydroxypseudooxynicotine from 6-hydroxynicotine via the catalysis of Hno and then added Pno to catalyze 6-hydroxypseudooxynicotine to obtain 6-hydroxy-3-succinoyl-semialdehyde-pyridine, which was identified using liquid chromatography-mass spectrometry (LC-MS) analysis (Fig. S2A). The prepared 6-hydroxy-3-succinoyl-semialdehyde-pyridine sample was further catalyzed by purified Ald using NAD^+^ as the coenzyme. The reaction product was determined by LC-MS analysis (Fig. S2B), which showed that Ald can effectively catalyze the dehydrogenation of 6-hydroxy-3-succinoyl-semialdehyde-pyridine into HSP.

### Ald can catalyze the reaction with SAP as a substrate.

**FIG 3:**
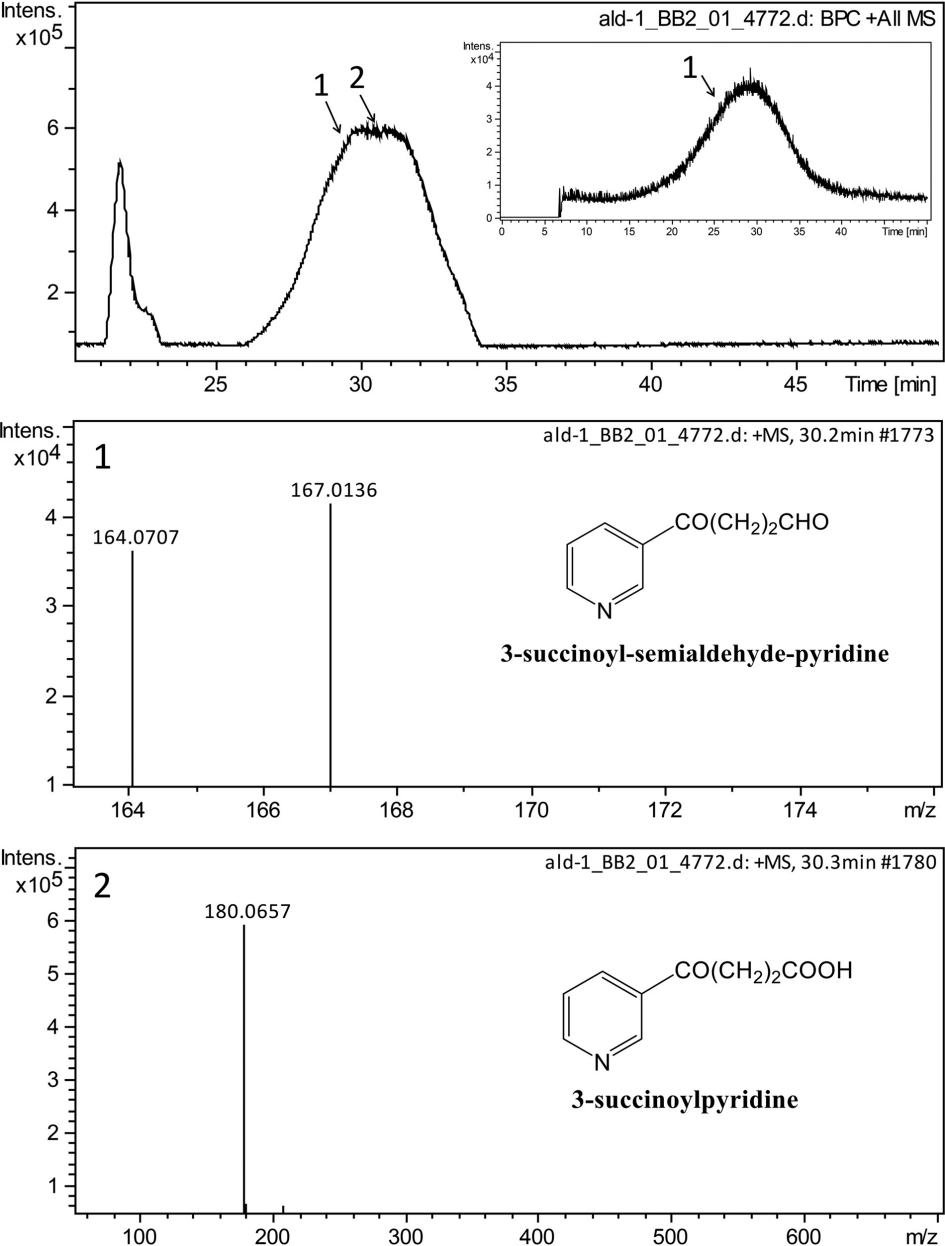
LC-MS profiles of Ald-catalyzed reaction with SAP as the substrate. The reaction mixture contained 50 mM Gly-NaOH buffer (pH 9.0), 0.4 mM SAP, 0.2 mM NAD^+^, and 1.2 μM Ald. (1) Mass spectrum of SAP (*m/z* 164.0707); the other peak (*m/z* 167.0136) is for the impurity. The inset shows the result of direct search using the molecular weight of 164.07. (2) Mass spectrum of 3-succinoylpyridine (*m/z* 180.0657), which was the product of the Ald-catalyzed reaction.

Because 6-hydroxy-3-succinoyl-semialdehyde-pyridine is difficult to obtain and quantify, the kinetic reaction properties of Ald cannot be determined. Therefore, we tested the reaction using SAP instead of 6-hydroxy-3-succinoyl-semialdehyde-pyridine as the substrate. NAD^+^ or NADP^+^ was used as the coenzyme in the tests, and their reduction was monitored at 340 nm. The reaction was found to be dependent on NAD, while NADP had no effect on the reaction (Fig. 2B). The UV absorption spectra (250 nm to 400 nm) showed a new peak with maximum absorption at 340 nm, confirming that NAD^+^ was reduced into NADH (Fig. 2C). Further LC-MS analysis revealed that 3-succinoylpyridine was the product of the Ald-catalyzed reaction with SAP as the substrate (Fig. 3).

**FIG 4:**
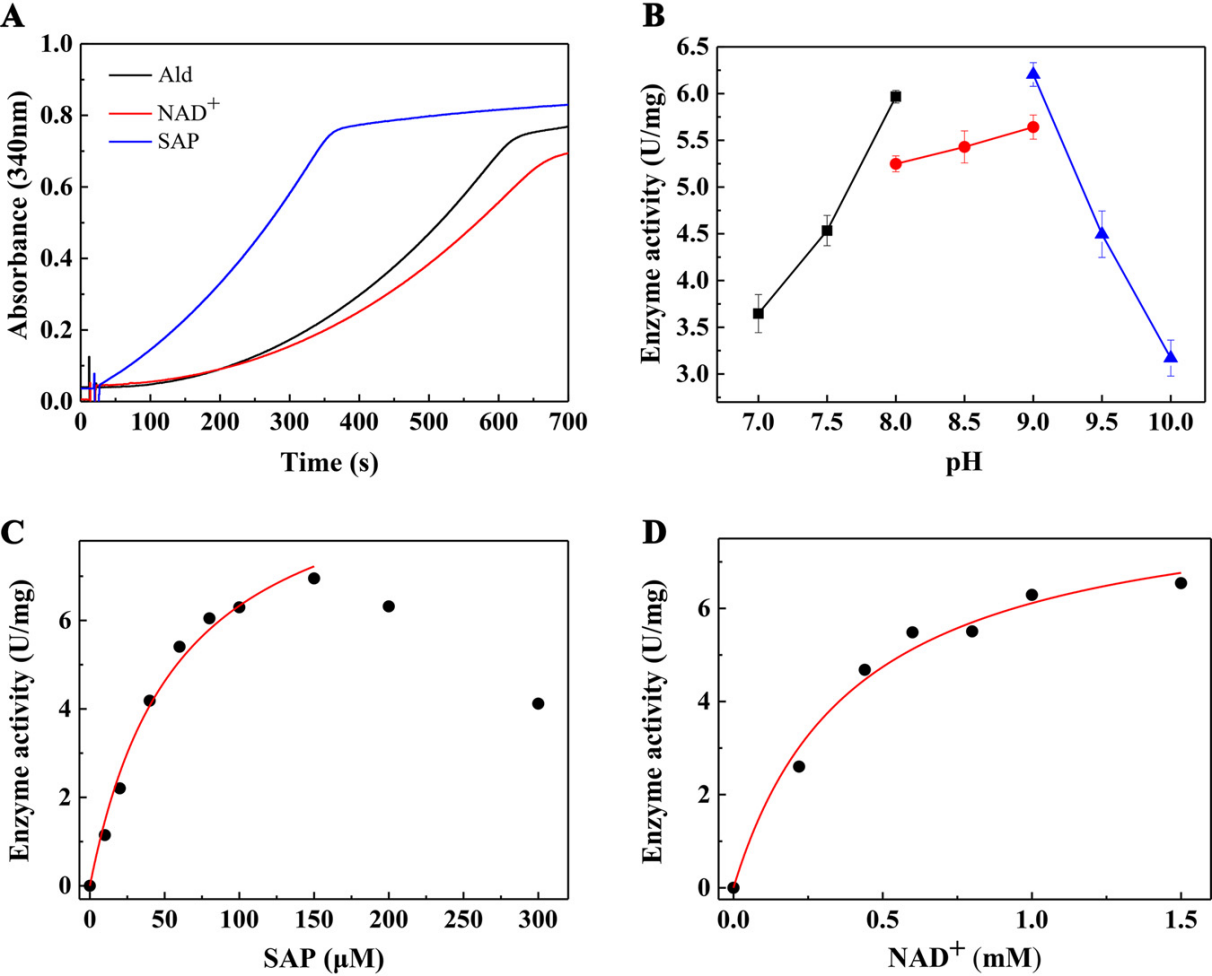
Kinetic differences of Ald-catalyzed reactions started with Ald, SAP, or NAD^+^ (A) and the determination of the reaction’s optimal pH (B) and apparent *K_m_* values for SAP (C) and NAD^+^ (D). The reaction mixture contained 50 mM Gly-NaOH buffer (pH 9.0), 0.1 mM SAP, 1 mM NAD^+^, 0.5 μM Ald, or as indicated. (A) Key: Ald, Ald was added to start the reaction after adding SAP and NAD^+^ to the reaction mixture; SAP, SAP was added to start the reaction after adding Ald and NAD^+^ to the mixture and incubating for 5 min; NAD^+^, NAD^+^ was added to start the reaction after adding Ald and SAP to the mixture and incubating for 5 min. For the determination of the optimal pH, 50 mM sodium phosphate buffer (pH 7.0/7.5/8.0), 50 mM Tris-HCl buffer (pH 8.0/8.5/9.0), and 50 mM Gly-NaOH buffer (pH 9.0/9.5/10.0) were used. For panels B to D, the reactions were started by adding 0.5 μM Ald.

The *ald* gene was also heterologously expressed with the C-terminally fused 6× His tag in E. coli BL21 cells (Fig. S3A). Ald with an N- or C-terminal 6× His tag (Ald-N and Ald-C, respectively) was used to catalyze SAP oxidation. It was found that the activity of Ald-N was 2.1 times higher than that of Ald-C (Fig. S3B), so Ald-N was used in subsequent experiments. Interestingly, the reaction presented a delay period when Ald was added to initiate the reaction in a mixture with NAD^+^ and SAP (Fig. 4A, black line). Thus, we tested the cases with the incubation of Ald and NAD^+^ or SAP for 5 min before adding SAP or NAD^+^ to initiate the reaction. In the reaction with the incubation of NAD^+^ and Ald first and starting the reaction with SAP, the delay period was shortened (Fig. 4A, blue line), and the activity of Ald was similar to that of the reaction directly started with Ald. However, a delay period still existed in the reaction with the incubation of SAP and Ald first and starting the reaction with NAD^+^, where the activity of Ald decreased slightly (Fig. 4A, red line). This may be caused by the reaction mechanism of aldehyde dehydrogenase, which is explained in detail in Discussion.

Because the incubation time of Ald and NAD^+^ was difficult to determine and Ald was not very stable under the conditions tested, the reactions in the subsequent experiments were still initiated by adding Ald immediately after adding NAD and SAP, and we selected the highest reaction rate, not the initial reaction rate, to calculate the enzyme activities. Thus, an assay to determine the Ald activity *in vitro* was developed, in which SAP and NAD^+^ were used as the substrate and coenzyme, respectively, and Ald was added to initiate the reaction. In this assay, the optimal pH for the Ald-catalyzed reaction was first measured, and then the *K_m_* values of SAP and NAD^+^ were determined. The optimal reaction pH was found to be 9.0 (Fig. 4B). The apparent *K_m_* of SAP is around 58.68 μM, and the corresponding *V*_max_ is around 10.05 U/mg (Fig. 4C). The apparent *K_m_* of NAD^+^ is around 0.41 mM, and the corresponding *V*_max_ is around 8.60 U/mg (Fig. 4D). Noticeably, there was a substrate inhibition in the reaction when the SAP concentration exceeded 150 μM.

### Disruption of the *ald* gene negatively affected the growth of A. tumefaciens S33 on nicotine.

**FIG 5:**
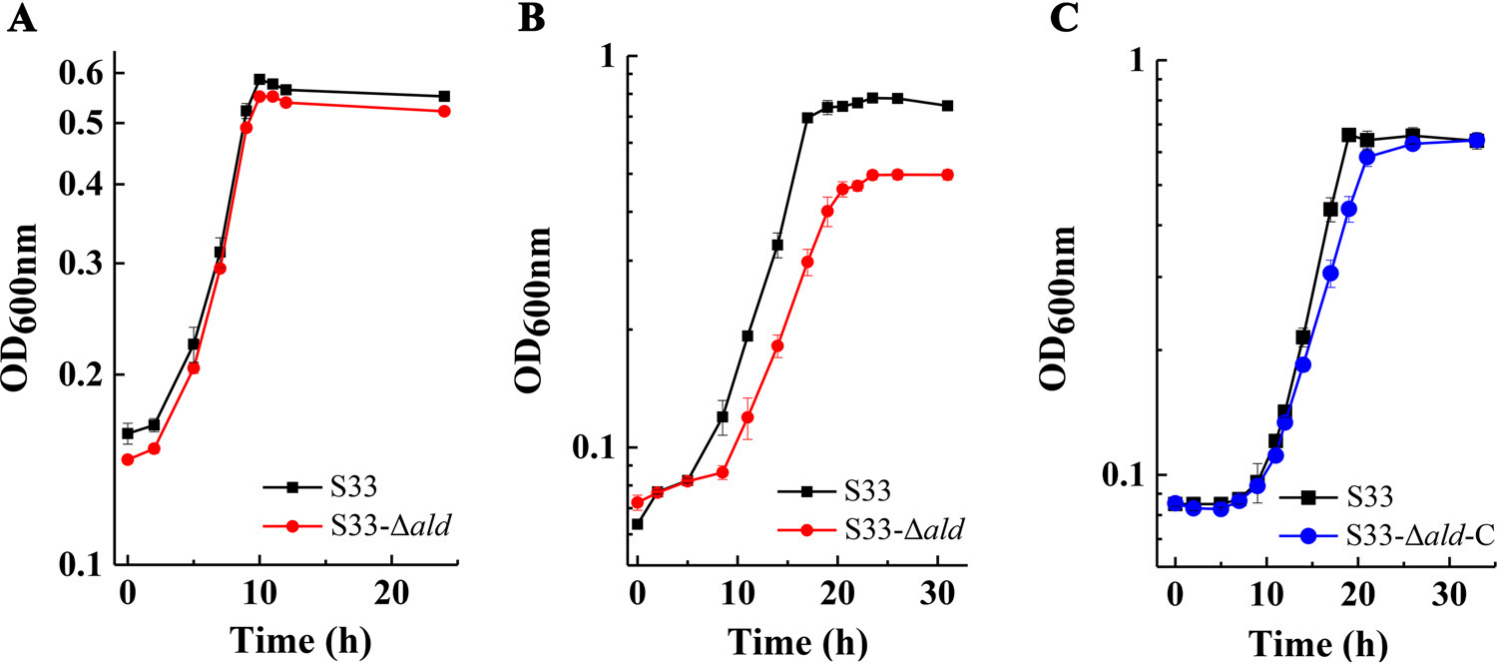
Growth of the wild-type A. tumefaciens S33, mutant strain S33-Δ*ald*, and complemented strain S33-Δ*ald*-C in HSP medium (A) and nicotine medium (B and C). HSP (0.5 g liter^−1^) or nicotine (1 g liter^−1^) was used as the sole source of carbon and nitrogen in the two media.

To verify the function of Ald *in vivo*, the *ald* gene of strain S33 was disrupted through homologous recombination, and then the mutant was complemented by introducing a full-length *ald* gene (Fig. S4). The growth of the wild-type strain, mutant strain S33-Δ*ald*, and complemented strain S33-Δ*ald*-C in an HSP or nicotine medium was determined. As shown by the results in Fig. 5, the growth of the mutant strain S33-Δ*ald* was similar to that of the wild-type strain in an HSP medium (Fig. 5A); however, the exponential growth of the mutant strain was slightly slower than that of the wild-type strain in the nicotine medium, and the final biomass was also less than that of the wild-type strain (Fig. 5B). After complementing the disruption of the *ald* gene, the growth and biomass of strain S33-Δ*ald*-C were similar to those of the wild-type strain (Fig. 5C). These results indicate that Ald plays an important role in the degradation of nicotine by A. tumefaciens S33 and functions in a step before HSP oxidation in the pathway.

### The broad substrate range of the Ald-catalyzed reaction.

The true substrate of Ald is a pyridine ring-containing aldehyde compound. To check the substrate range of Ald, we selected several aldehyde compounds for kinetic determination. Benzaldehyde was selected as a substrate among the aldehydes with benzene rings. With an optimal pH of 9.0 for the reaction (Fig. S5A), Ald presented an apparent *K_m_* of around 0.18 mM for benzaldehyde and a *V*_max_ of around 7.01 U/mg (Fig. S5B); the apparent *K_m_* of NAD^+^ is around 0.47 mM, and the corresponding *V*_max_ is 6.47 U/mg (Fig. S5C). Similarly, when the benzaldehyde reaction concentration exceeded 400 μM, the reaction showed substrate inhibition. Furfural was selected as the substrate among the aldehydes with a furan ring. With an optimal pH of 8.5 for the reaction (Fig. S6A), the apparent *K_m_* of furfural was determined to be 0.43 mM, and the corresponding *V*_max_ was 5.24 U/mg (Fig. S6B); the apparent *K_m_* of NAD^+^ was 0.60 mM, and the corresponding *V*_max_ was 5.26 U/mg (Fig. S6C). Acetaldehyde was selected as the substrate among the short-chain aldehydes. The apparent *K_m_* of acetaldehyde was 0.47 mM, and the corresponding *V*_max_ was 3.75 U/mg (Fig. S7A); the apparent *K_m_* of NAD^+^ was 0.41 mM, and the corresponding *V*_max_ was 2.87 U/mg (Fig. S7B). The results indicate that the Ald in strain S33 has a broad substrate range.

### Biotransformation of furfural by the recombinant E. coli_Ald cells.

**FIG 6:**
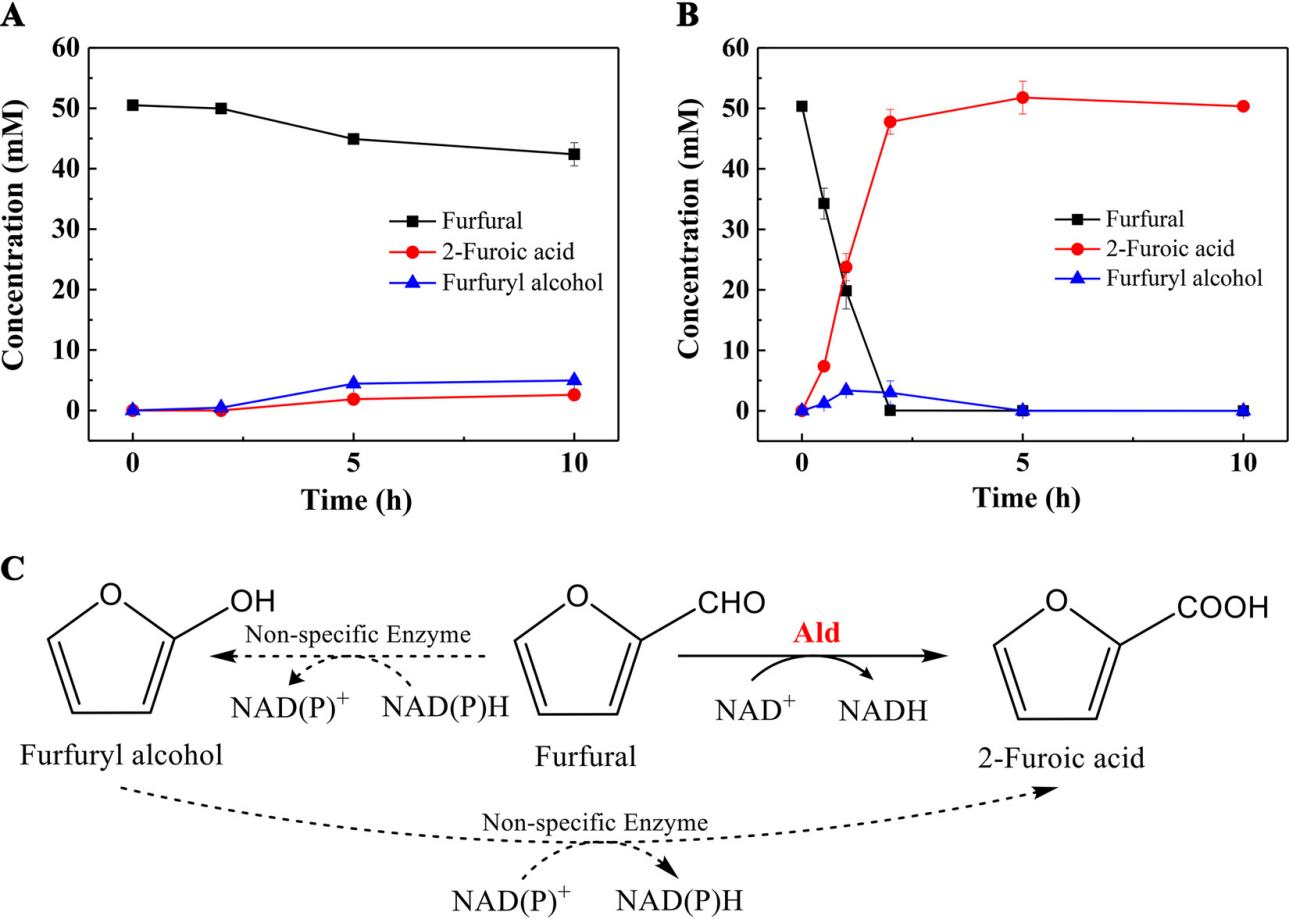
Progress curve of the biocatalytic oxidation of furfural. (A) Control group, where the whole cells of the E. coli BL21 harboring the blank pET-Duet vector were used. (B) Experimental group, where the whole cells of the E. coli BL21 harboring the recombinant pET-Duet-Ald were used. (C) Diagram of the biocatalytic oxidation of furfural to 2-furoic acid. The dashed arrows indicate the nonspecific intracellular reactions, and the solid arrow indicates the formation of 2-furoic acid catalyzed by Ald.

The oxidation of furfural by Ald was also identified using LC-MS analysis (Fig. S8), which proved that Ald did indeed catalyze the *in vitro* dehydrogenation of furfural into 2-furoic acid. Considering the value of the furan derivatives in the production of biocommodities, we tried to use the whole cells of the recombinant E. coli harboring the *ald* gene as the catalyst to test the possibility of biotransformation of furfural into 2-furoic acid. As shown by the results in Fig. 6, compared with the control with E. coli cells harboring a blank plasmid vector, the recombinant E. coli_Ald cells could efficiently transform the substrate furfural into 2-furoic acid. Furfural (50 mM) was completely converted in 2 h to produce about 47.7 mM 2-furoic acid, with a yield of 95.4% at pH 8.0 and 30°C (Fig. 6B). The specific reaction rate of the recombinant cells reached 0.032 mmol min^−1^ g dry cells^−1^. In addition, furfuryl alcohol was also produced as a by-product, which might be catalyzed by the intrinsic reductase/dehydrogenase of the host cells (Fig. 6C). Furfuryl alcohol could be slowly reoxidized to 2-furoic acid, which is a necessary step for the conversion of furfural into 2-furoic acid (29). The product yield reached 99% after 10 h of reaction. In contrast, in the control, only 16.2% of furfural was transformed in 24 h, indicating that the heterologous expression of Ald in E. coli mainly functions in the transformation of furfural into 2-furoic acid.

## DISCUSSION

Our previous transcriptomic analysis found that the expression level of the *ald* gene in the nicotine-degrading gene cluster of A. tumefaciens strain S33 was upregulated in nicotine medium in the same pattern as other core nicotine-degrading genes (19). It was predicted that the *ald* gene is involved in nicotine catabolism. In this study, we experimentally verified the function of Ald to catalyze the dehydrogenation of 6-hydroxy-3-succinoyl-semialdehyde-pyridine into HSP. This conclusion was drawn based on the following results: (i) the purified Ald catalyzed 6-hydroxy-3-succinoyl-semialdehyde-pyridine into HSP, with NAD^+^ as its coenzyme; (ii) SAP can function as the substrate of Ald instead of 6-hydroxy 3-succinoyl-semialdehyde-pyridine for kinetic characterization, and with SAP Ald has a sufficient reaction rate and *K_m_* values for the substrates to participate in the nicotine degradation *in vivo*; and (iii) the disruption of the *ald* gene led to slower growth and lower biomass of strain S33 on nicotine but not on HSP.

In our experiments, aggregation and precipitation of the purified recombinant Ald occurred, which blocked the way to characterizing its properties and identifying its function. We tried many conditions for the purification and storage of Ald *in vitro* and found that a low-salt and alkaline environment could alleviate its aggregation and precipitation. Thus, biochemical tests could be conducted under these conditions. The *in vitro* oxidation of 6-hydroxy-3-succinoyl-semialdehyde-pyridine (obtained by coupling Hno and Pno reactions from 6-hydroxynicotine) into HSP, catalyzed by Ald, was conclusively identified by LC-MS (Fig. S2). Since it was difficult to obtain purified 6-hydroxy-3-succinoyl-semialdehyde-pyridine, SAP was used instead for kinetic tests. The LC-MS analysis and kinetic tests showed that Ald could catalyze the dehydrogenation of SAP into 3-succinoylpyridine with NAD^+^ as its coenzyme (Fig. 3). In the reaction, Ald was strictly dependent on NAD^+^, and NADP^+^ could not substitute for NAD^+^ (Fig. 2). In addition, the reaction rates with SAP as a substrate and the *K_m_* values for the substrates were comparable to those of other enzymes involved in nicotine degradation in strain S33 (22); these findings are sufficient to support Ald as participating in nicotine degradation *in vivo*. Furthermore, the disruption of the *ald* gene in strain S33 had a negative effect on the growth of strain S33 in nicotine medium but not in HSP medium (Fig. 5), suggesting that the nicotine degradation was interrupted before HSP oxidation in the pathway, leading to the accumulation of the intermediates and the failure of strain S33 to fully utilize nicotine. In nicotine medium, the exponential growth rate of the mutant strain was only slightly slower than that of the wild-type strain, which might be due to the existence of multiple aldehyde dehydrogenases in strain S33 and their properties in a wide range of substrates. Previous transcriptomic analysis showed that the genes for aldehyde dehydrogenase (AWN88_00395 and AWN88_04025) also had high transcription levels in nicotine medium (19), which may alleviate the effects caused by the disruption of the *ald* gene. These results indicate that Ald functions in catalyzing the oxidation of 6-hydroxy-3-succinoyl-semialdehyde-pyridine into HSP in the nicotine degradation pathway in strain S33.

We found that there was a delay period after Ald was added into the reaction mixture to start the reaction. This is because aldehyde dehydrogenase usually catalyzes the reaction following an ordered BiBi steady-state kinetic mechanism (31, 32). The Ald from strain S33 should catalyze the reaction in the same way (Fig. S9). The reaction begins with the binding of NAD^+^ to the catalytic cavity of the enzyme, which induces the deprotonation of the reactive cysteine; then, the nucleophilic attack of cysteine on the aldehyde results in the formation of the thiohemiacetal intermediary. The hydrogen of the thiohemiacetal intermediary is further transferred to the coenzyme to form the thioester adduct, which is hydrolyzed by water molecules to release the product. Finally, the reduced coenzyme is released from the enzyme. When Ald is exposed to NAD^+^ and SAP at the same time, it first reacts with coenzyme NAD^+^ and then combines with SAP, forming a delay period. Therefore, when Ald was incubated with NAD^+^ first and then SAP was added to start the reaction, the delay period was significantly shortened (Fig. 4A). Due to the instability of Ald in our assays, we still added NAD^+^ and SAP first, followed by Ald to start the reaction.

We also found that Ald carrying a His tag at the N terminus was more stable and had a higher activity than that carrying a His tag at the C terminus (Fig. S3B). According to the known structures of various aldehyde dehydrogenases (33–36), the sequence close to the C terminus of some aldehyde dehydrogenases is related to the dimer formation. The His tag at the C terminus affects the binding of the dimer, resulting in instability and low activity of the enzyme. We also analyzed and predicted the NAD^+^ binding region and SAP catalytic region of Ald by aligning Ald with multiple reported aldehyde dehydrogenase protein sequences in the literature (Fig. S10). In addition, when SAP or benzaldehyde was used as a substrate, it was found that high substrate concentrations could inhibit the activity of Ald (Fig. 4C; Fig. S5B). In previous reports, betaine aldehyde dehydrogenase (BADH) also presented a substrate or substrate analog inhibition effect (37–40). It was found that the substrate inhibition of SAP or benzaldehyde was uncompetitive substrate inhibition (Fig. S11), similar to the case of BADH (39). The substrate with a high concentration would combine with the NADH-enzyme complex produced in the last reaction cycle to form an NADH-enzyme-substrate ternary complex, which caused the slow release of NADH. This might explain the high concentrations of SAP and benzaldehyde’s inhibition of Ald activity in strain S33.

Furfural and acetaldehyde did not display this phenomenon, possibly because of the large difference in molecular structure compared to SAP, as indicated by their higher apparent *K_m_* values. Moreover, it was found that Ald from strain S33 had a lower activity in Tris-HCl buffer than in phosphate and Gly-NaOH buffers when using SAP, benzaldehyde, or furfural as the substrate, and strain S33 Ald was completely inactivated in the case of acetaldehyde (Fig. 4B; Fig. S5A and S6A), which is also similar to the case of BADH. It was reported that an appropriate concentration of K^+^ or Na^+^ was required to maintain the stability and activity of BADH, but not with a concentration above 500 mM, which might explain the low activity of Ald in Tris-HCl buffer and its poor stability in a high-salt environment (37–41).

The Ald of strain S33 also had high activity on benzaldehyde, furfural, and acetaldehyde, demonstrating its versatility for different substrates (Fig. S5 to S7). To test the possible application of Ald in industrial biocatalysis, we used the whole cells of the recombinant E. coli_Ald to catalyze furfural into 2-furoic acid (Fig. 6). It was found that furfural could be efficiently converted into 2-furoic acid with recombinant E. coli_Ald cells as the catalyst. The results suggest that Ald can be used for the catalysis of bio-based furan compounds. In the future, the recombinant strain can be further modified to improve its efficiency and allow it to be used in the production of different valuable chemicals.

In summary, we investigated the function of Ald in the nicotine degradation of A. tumefaciens S33 through mass spectrum analysis, biochemical assay, and gene disruption. Although the nonhydroxylated analog SAP was used instead of its real substrate 6-hydroxy-3-succinoyl-semialdehyde-pyridine in some *in vitro* experiments, the results were expected to be identical to those using the true substrate. Thus, we could conclude that Ald is responsible for the dehydrogenation of 6-hydroxy-3-succinoyl-semialdehyde-pyridine into HSP in nicotine degradation in strain S33. Unlike SAPD of Pseudomonas sp. strain HZN6, which uses NADP^+^ as a coenzyme, Ald is NAD^+^ specific. The Ald-catalyzed reaction provides the first NADH molecule for the growth of strain S33 with nicotine as the sole carbon and nitrogen source (other NADH molecules are generated after the metabolism enters the TCA cycle). The identification of this enzyme solves the problem of the last unknown enzyme in this hybrid pathway of nicotine degradation in strain S33 and helps to understand the electron transfer and redox metabolism in the oxidative degradation of nicotine. In addition, Ald has a broad substrate range, including furfural, benzaldehyde, and acetaldehyde. Based on this feature, the recombinant E. coli_Ald cells were used as the catalyst to transform furfural into 2-furoic acid, extending the application value of Ald in the catalysis of bio-based furan compounds. This study provides new knowledge of the biochemical mechanism in the hybrid pathway of nicotine degradation in A. tumefaciens S33 and preliminary exploration of the application of Ald in biocatalysis.

## MATERIALS AND METHODS

### Bacterial strains, culture conditions, and biochemicals.

A. tumefaciens strain S33 was deposited with accession number CCTCC AB 2016054 at the China Type Culture Collection (CCTCC). Nicotine (1 g liter^−1^) was used as the sole source of carbon and nitrogen to grow strain S33 at 30°C. E. coli strain DH5α and E. coli strain BL21(DE3) were cultured in LB medium at 37°C. NAD^+^ and NADP^+^ were purchased from Sigma-Aldrich (Shanghai, China), 6-hydroxynicotine and SAP were purchased from Toronto Research Chemicals, Inc. (Toronto, Canada), and benzaldehyde, furfural, and acetaldehyde were purchased from Macklin (Shanghai, China). Carbenicillin (25 mg liter^−1^) and kanamycin (50 mg liter^−1^) were required to culture the E. coli cells.

### Heterologous expression and purification of Ald.

Using the genomic DNA of A. tumefaciens S33 as the template, the *ald* gene (locus tag AWN88_01340) was amplified. The following primers were used for N-terminal ligation of a 6× His tag: 5′-CGCGGATCCGATGACAAACTTCAACATGCTAATAAACG-3′ (forward, BamHI recognition site is underlined) and 5′-CCCAAGCTTTTAAGCTGCGTTGAGGATTTGTAC-3′ (reverse, HindIII recognition site is underlined). The following primers were used for C-terminal ligation of a 6× His tag: 5′-CGCGGATCCGATGACAAACTTCAACATGCTAATAAACG-3′ (forward, BamHI recognition site is underlined) and 5′-CCCAAGCTTAGCTGCGTTGAGGATTTGTACGCGT-3′ (reverse, HindIII recognition site is underlined). The *ald* gene was cloned into plasmids pETDuet (N-terminal His tag) and pET24b(+) (C-terminal His tag), and the constructs were transformed into E. coli BL21(DE3) cells. The cells were cultured at 37°C in LB medium containing 25 mg liter^−1^ carbenicillin or 50 mg liter^−1^ kanamycin to an optical density (OD) of 0.6 at 600 nm. Then, 0.3 mM IPTG (isopropyl-β-d-thiogalactopyranoside) was added, and the cells were grown at 16°C for 20 h. The cells were collected by centrifugation and resuspended in lysis buffer (20 mM sodium phosphate, 0.25 M NaCl, 1 mM dithiothreitol [DTT], 1 mM phenylmethylsulfonyl fluoride [PMSF], and 10% glycerol, pH 8.0). After destroying the cells through using a low-temperature and ultra-high-pressure continuous flow cell using JN-02C (JNBIO, Guangzhou, China), the sample was centrifuged at 35,328 × *g* for 1 h at 4°C. The supernatant was loaded on a 5-ml HisTrap column (GE Healthcare, Little Chalfont, Buckinghamshire, UK) previously equilibrated with a binding buffer (20 mM sodium phosphate, 0.25 M NaCl, and 1 mM DTT, pH 8.0). The target protein was eluted at 0.1 M imidazole using a stepwise elution gradient. The protein was washed with 50 mM sodium phosphate buffer (pH 8.0) and stored at 4°C after concentration.

### Assay of Ald activity.

By coupling the Hno and Pno reactions, 6-hydroxy-3-succinoyl-semialdehyde-pyridine was prepared from 6-hydroxynicotine. Briefly, 6-hydroxynicotine was catalyzed by Hno to generate 6-hydroxypseudooxynicotine and 6-hydroxy-*N*-methylmyosmine, and 6-hydroxypseudooxynicotine was further catalyzed by Pno to produce 6-hydroxy-3-succinoyl-semialdehyde-pyridine, which was used as a substrate for Ald. The preparation of Hno and Pno and the reaction conditions were as described previously (15–17, 20). The reaction mixture contained 50 mM Gly-NaOH buffer (pH 9.0), 4.4 mM 6-hydroxynicotine, 30 mM NaCl, 0.5 mM PMS (phenazine methosulfate), 0.6 mM DCPIP (2,6-dichlorophenolindophenol), 4.3 μM Hno, and 0.9 μM Pno. The reaction product was determined using LC-MS analysis. Then, the reaction mixture with 6-hydroxy-3-succinoyl-semialdehyde-pyridine was supplemented with 2 mM NAD^+^ and 1 μM Ald to start the Ald-catalyzed reaction.

To measure the activity of Ald, a reaction mixture was made with 50 mM Gly-NaOH (pH 9.0), 1 mM NAD^+^, Ald, and aldehyde compound as indicated. The aldehyde compounds tested were SAP, benzaldehyde, furfural, and acetaldehyde. The reaction was performed at 30°C and monitored at 340 nm (NADH, ε = 6.22 mM^−1^ cm^−1^). One unit (U) was defined as the production of 1 μmol of NADH per minute. To measure the optimal pH for the Ald reactions, 50 mM sodium phosphate buffer (pH 7.0/7.5/8.0), 50 mM Tris-HCl buffer (pH 8.0/8.5/9.0), and 50 mM Gly-NaOH buffer (pH 9.0/9.5/10.0) were used. Three replicates were performed, and the mean values are shown with the standard deviations indicated by error bars.

### Cultivation of recombinant E. coli_Ald cells and biocatalytic oxidation of furfural.

The recombinant E. coli_Ald cells and E. coli cells harboring a blank plasmid vector were cultured at 37°C in LB medium containing 25 mg liter^−1^ carbenicillin to an OD of 0.6 at 600 nm. Then, 0.3 mM IPTG was added, and the cells were grown at 30°C for 20 h. The cells were collected by centrifugation and washed twice with 20 mM sodium phosphate. Typically, 50 ml of phosphate buffer (50 mM, pH 8.0) containing 50 mM furfural, 0.5 M CaCO_3_, and 17.1 mg dry cells ml^−1^ (one OD unit at 600 nm = 0.38 g dry cells liter^−1^) was incubated at 30°C and 200 rpm. Aliquots were withdrawn from the reaction mixtures at specified time intervals. The contents of the substrate and products were quantified by high-performance liquid chromatography (HPLC) analysis.

### Analytical methods.

The protein concentration was measured using Bradford analysis with bovine serum albumin as the standard (42). Enzymatic assays were carried out in quartz cuvettes (1-cm light path) with a total of 500 μl of the reaction mixture, and the reactions were monitored by using a UV-visible light Ultrospec 2100 pro spectrophotometer (GE Healthcare, USA). The relative molecular mass of the protein was determined by gel filtration on a GE Superdex G200 column (10 mm by 300 mm) as described previously (15).

The reaction products of Ald with different substrates were analyzed and identified using LC-MS (15). The reaction mixtures were mixed with equal volumes of ethanol. After freezing at −20°C for 1 h, the mixture was centrifuged at 18,000 × *g* and 4°C for 5 min. The supernatant was used for injection. HPLC-tandem MS (MS/MS) was performed on a rapid separation liquid chromatography system (UltiMate3000 ultra-high-pressure liquid chromatography [UHPLC] instrument; Dionex) coupled with an electrospray ionization-quadrupole time of flight (ESI-Q-TOF) mass spectrometer (Impact HD; Bruker Daltonics). Chromatographic separations were performed on a Zorbax Eclipse XDB-C_18_ column (250 mm by 4.6 mm, 5-μm particle size; NanoChrom) at 30°C, with a mobile phase system containing 8 mM formic acid in Milli-Q filtered water (A) and methanol (B). A gradient program of 95% to 5% A plus 5% to 95% B for 0 to 50 min at a flow rate of 0.7 ml min^−1^ was applied. The injection volume was 5 μl. To determine the products of Ald-catalyzed furfural, in a mobile phase system containing 0.4% ammonium acetate (pH 3.5) (A) and acetonitrile (B) (90:10, vol/vol), a gradient program was applied at a flow rate of 0.6 ml min^−1^ for 40 min with an injection volume of 5 μl.

The biocatalytic oxidation of furfural by the whole cells of recombinant E. coli was determined using HPLC (30). The collected cells were boiled in a water bath for 10 min and centrifuged at 18,360 × *g* for 10 min at 4°C, and the supernatant was diluted 10 times with the mobile phase. A Zorbax Eclipse XDB-C_18_ column (250 mm by 4.6 mm, 5-μm particle size; NanoChrom) was used. The mobile phase was methanol and Milli-Q filtered water (10:90, vol/vol), which contained 0.4% (NH_4_)_2_SO_4_ solution with a pH of 3.5. The flow rate was set to 0.6 ml min^−1^, and the injection volume was 10 μl. The detection wavelengths were set at 260 nm to detect furfural and 2-furoic acid and at 216 nm for furfuryl alcohol. The column temperature was 30°C. Standard curves of furfural, 2-furoic acid, and furfuryl alcohol were prepared for quantification.

### Disruption of the *ald* gene.

The *ald* gene in the genome of A. tumefaciens S33 was disrupted by homologous recombination using the suicide plasmid vector pJQ200SK, and then the mutant was complemented with pBBR1MCS-5 harboring the full-length *ald* gene as described previously (20). The primer sequences for *ald* gene disruption were as follows: 5′-CGCGGATCCATGACAGAAAAGATATATGATGC-3′ (primer A, BamHI recognition site is underlined), 5′-ACGCCGGGCGGAAAAATACCTTCGGCAAGTCGCCCCAGCTCT-3′ (primer B), 5′-AGGTATTTTTCCGCCCGGCGTGGACGTCAACCTTGGCCCTCT-3′ (primer C), and 5′-CGCGGGCCCTTAAGCTGCGTTGAGGATTTGT-3′ (primer D, ApaI recognition site is underlined). The primer sequences for *ald* gene complementation were as follows: 5′-CCCAAGCTTGATGACAAACTTCAACATGCTAGCT-3′ (primer F, HindIII recognition site is underlined) and 5′-CGCGGATCCTTAAGCTGCGTTGAGGATTTGTAC-3′ (primer R, BamHI recognition site is underlined). To compare the effects of gene disruption and complementation on cell growth, the mutant strain S33-Δ*ald*, complemented strain S33-Δ*ald*-C, and wild-type strain A. tumefaciens S33 (the control) were grown in HSP (0.5 g liter^−1^) or nicotine (1 g liter^−1^) medium at 30°C.
