# Supplementary material for: An NAD-Specific 6-Hydroxy-3-Succinoyl-Semialdehyde-Pyridine Dehydrogenase from Nicotine-Degrading Agrobacterium tumefaciens Strain S33
Source: Microbiol Spectr. 2021 Aug 11;9(1):10.1128/spectrum.00924-21. doi: 10.1128/spectrum.00924-21 (PMC8552603; doi:10.1128/spectrum.00924-21)
Supplement: SUPPLEMENTAL FILE 1 — Supplemental material. Download SPECTRUM00924-21_Supp_1_seq8.pdf, PDF file, 1.5 MB [file spectrum00924-21_supp_1_seq8.pdf]

Supplemental materials

An NAD-specific 6-hydroxy-3-succinoyl-semialdehyde-pyridine dehydrogenase from nicotine-degrading *Agrobacterium tumefaciens* S33

Jinmeng Shang,[a] Xia Wang,[a] Meng Zhang,[a] Lexin Li,[a] Rufei Wang,[a] Haiyan Huang,[b] Shuning Wang[a#]

State Key Laboratory of Microbial Technology, Microbial Technology Institute, Shandong University, Qingdao 266237, People's Republic of China[a]; Institute of Basic Medicine, Shandong First Medical University & Shandong Academy of Medical Science, Jinan 250062, People's Republic of China[b]

Running head: Ald plays a key role in nicotine degradation

[#]Address correspondence to Shuning Wang, shuningwang@sdu.edu.cn.

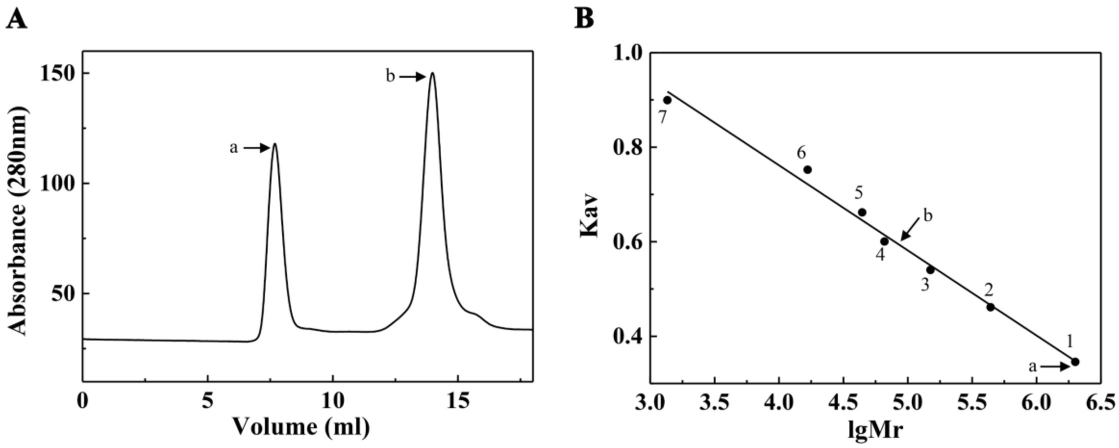

FIG S1 Determination of the relative molecular mass of Ald-N by gel filtration (A and B). (A) The elution curve for purified Ald-N. **a**, 7.682 ml; **b**, 13.977 ml. (B) 1. Dextran Blue 2000 (2,000 kDa); 2. Ferritin from equine spleen (440 kDa); 3. γ-Globulins from bovine blood (150 kDa); 4. Bovine serum albumin (66 kDa); 5. Albumin from chicken egg white (44.3 kDa); 6. Myoglobin (16.7 kDa); and 7. Vitamin B12 (1,360 Da). The molecular mass of the protein eluted as peak **b** is determined to be 110 kDa, and the molecular mass of the protein eluted as peak **a** is >2,000 kDa.

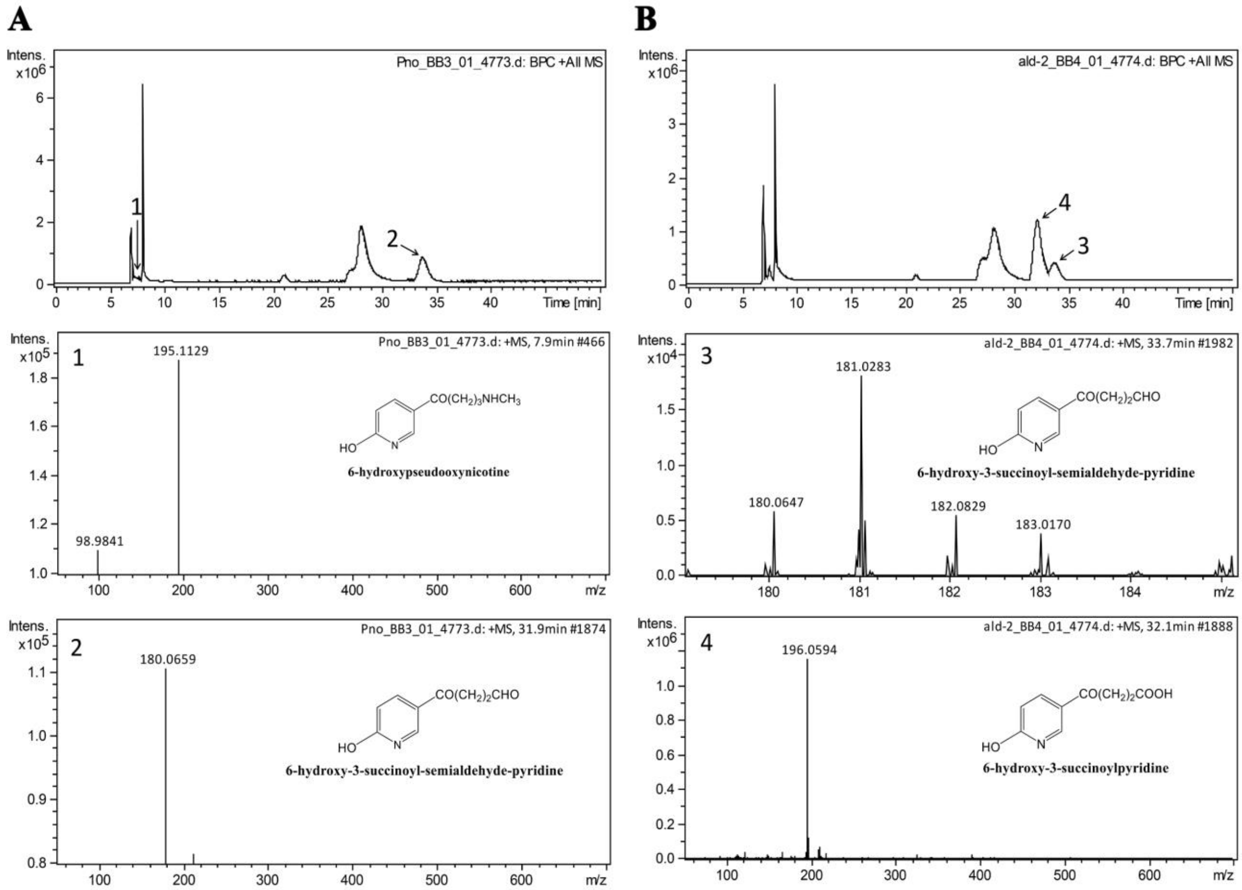

FIG S2 LC-MS profiles of the reactions catalyzed by Hno-Pno (A) and Ald (B). (A) LC-MS profiles of the reaction catalyzed by Pno and Hno with 6-hydroxynicotine as a substrate, the reaction mixture contained 50 mM Gly-NaOH buffer (pH 9.0), 0.8 mg/ml 6-hydroxynicotine, 30 mM NaCl, 0.5 mM PMS, 0.6 mM DCPIP, 4.3 μM Hno, and 0.9 μM Pno. The reaction was carried out on the basis of the previous experiments (Shang et al. 2021). (1) Mass spectrum of the product 6-hydroxypseudooxynicotine (*m/z* 195.1129) from 6-hydroxynicotine oxidation catalyzed by Hno. (2) Mass spectrum of the product 6-hydroxy-3-succinoyl-semialdehyde-pyridine (*m/z* 180.0659) of Pno-catalyzed reaction. (B) LC-MS profiles of the reaction catalyzed by Ald using the product 6-hydroxy-3-succinoyl-semialdehyde-pyridine from Hno-Pno reaction as a

substrate. The reaction mixture contained 50 mM Gly-NaOH buffer (pH 9.0), Hno-Pno reaction solution, 2 mM NAD^+, and 1 μM Ald. (3) Mass spectra of the excess substrate 6-hydroxy-3-succinoyl-semialdehyde-pyridine (*m/z* 180.0647), other peaks (*m/z* 181.0283, *m/z* 182.0829, and *m/z* 183.0170) are for impurities. (4) Mass spectrum of 6-hydroxy-3-succinoylpyridine (*m/z* 196.0594), which was the product of Ald-catalyzed reaction.

Reference

Shang J, Wang X, Zhang M, Wang R, Zhang C, Huang H, Wang S. 2021. Rid enhances the 6-hydroxypseudooxynicotine dehydrogenase reaction in nicotine degradation by *Agrobacterium tumefaciens* S33. Appl Environ Microbiol 87: e02769-20.

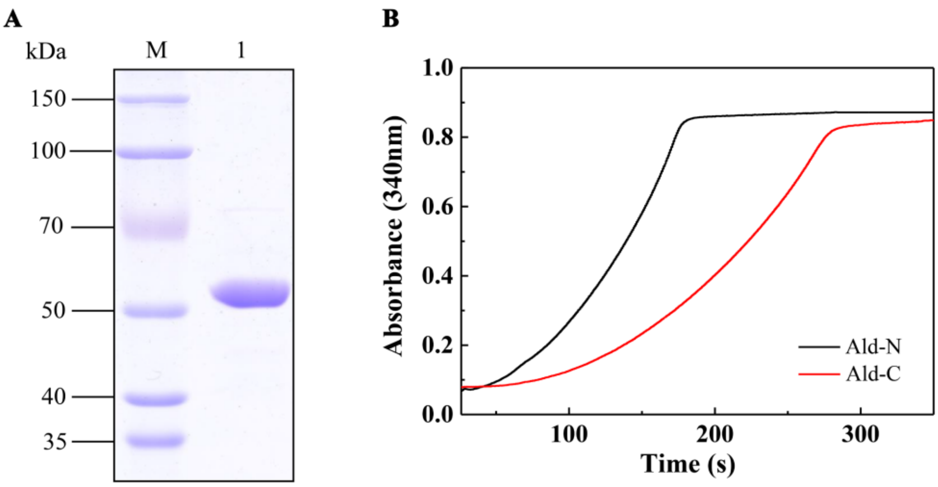

FIG S3 (A) SDS-PAGE analysis of purified His-tagged recombinant Ald-C. (B) The difference between Ald-N and Ald-C catalyzed reactions with NAD^+ as a cofactor and SAP as a substrate. The reaction mixture contains 50 mM Gly-NaOH buffer (pH 9.0), 0.1 mM SAP, 1 mM NAD^+, and 0.5 μM Ald-N/Ald-C.

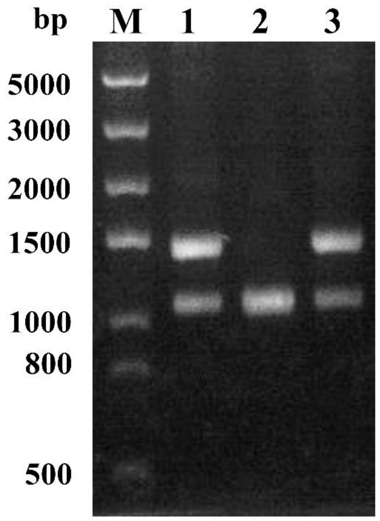

FIG S4 Disruption of the *ald* gene in *A. tumefaciens* S33 and its complementation.1, homologous single crossover strain, where the long band (1,398 bp) shows the PCR product of full length of the *ald* gene, and the short band (1,044 bp) shows the PCR product of partial deleted *ald* gene; 2, homologous double-crossover strain, where only the PCR product of the partial deleted *ald* gene (1,044 bp) was detected, indicating that *ald* gene is disrupted; 3, complementation strain, where the long band (1,398 bp) shows the PCR product amplified from the complementation plasmid pBBR1MCS-5 containing the full length of *ald* gene, and the short band (1,044 bp) shows the PCR product of partial deleted *ald* gene on the chromosome.

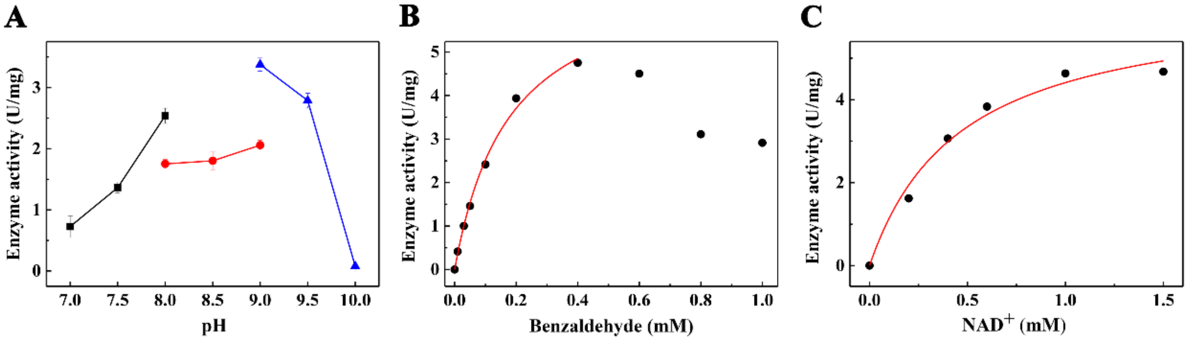

FIG S5 The determination of the reaction optimal pH (A) and K_m values for benzaldehyde (B) and NAD^+ (C) catalyzed by Ald with benzaldehyde as a substrate. The reaction mixture contains 50 mM Gly-NaOH buffer (pH 9.0), 0.4 mM benzaldehyde, 1 mM NAD^+, 0.5 μM Ald, or as indicated. For determination of optimal pH, the 50 mM sodium phosphate buffer (pH 7.0/7.5/8.0), 50 mM Tris-HCl buffer (pH 8.0/8.5/9.0), and 50 mM Gly-NaOH buffer (pH 9.0/9.5/10.0) were used.

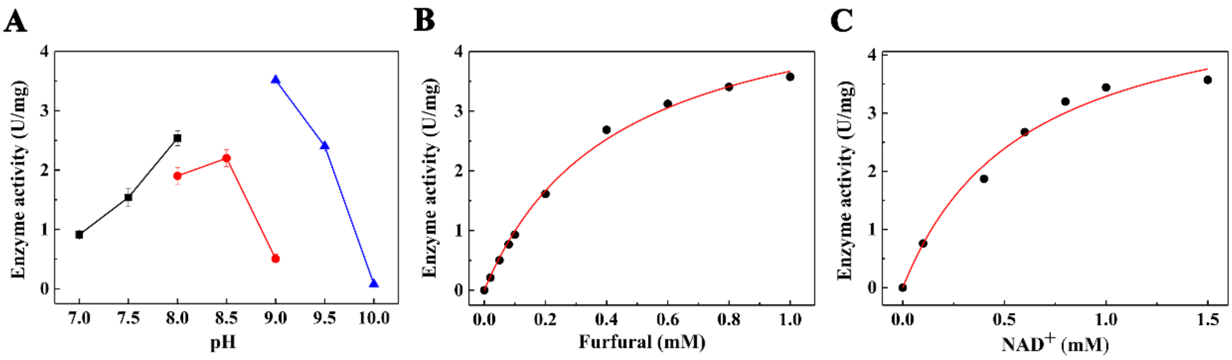

FIG S6 The determination of the reaction optimal pH (A) and K_m values for furfural (B) and NAD^+ (C) catalyzed by Ald with furfural as a substrate. The reaction mixture contains 50 mM Gly-NaOH buffer (pH 9.0), 1 mM furfural, 1 mM NAD^+, 1.0 μM Ald, or as indicated. For determination of optimal pH, the 50 mM sodium phosphate buffer (pH 7.0/7.5/8.0), 50 mM Tris-HCl buffer (pH 8.0/8.5/9.0), and 50 mM Gly-NaOH buffer (pH 9.0/9.5/10.0) were used.

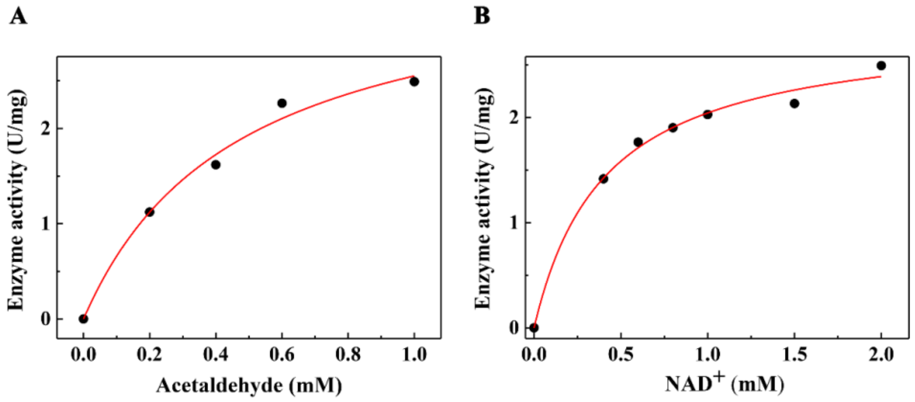

FIG S7 The determination of K_m values for acetaldehyde (A) and NAD^+ (B) catalyzed by Ald with acetaldehyde as a substrate. The reaction mixture contains 50 mM Gly-NaOH buffer (pH 9.0), 1.0 mM acetaldehyde, 1.0 mM NAD^+, 1 μM Ald, or as indicated.

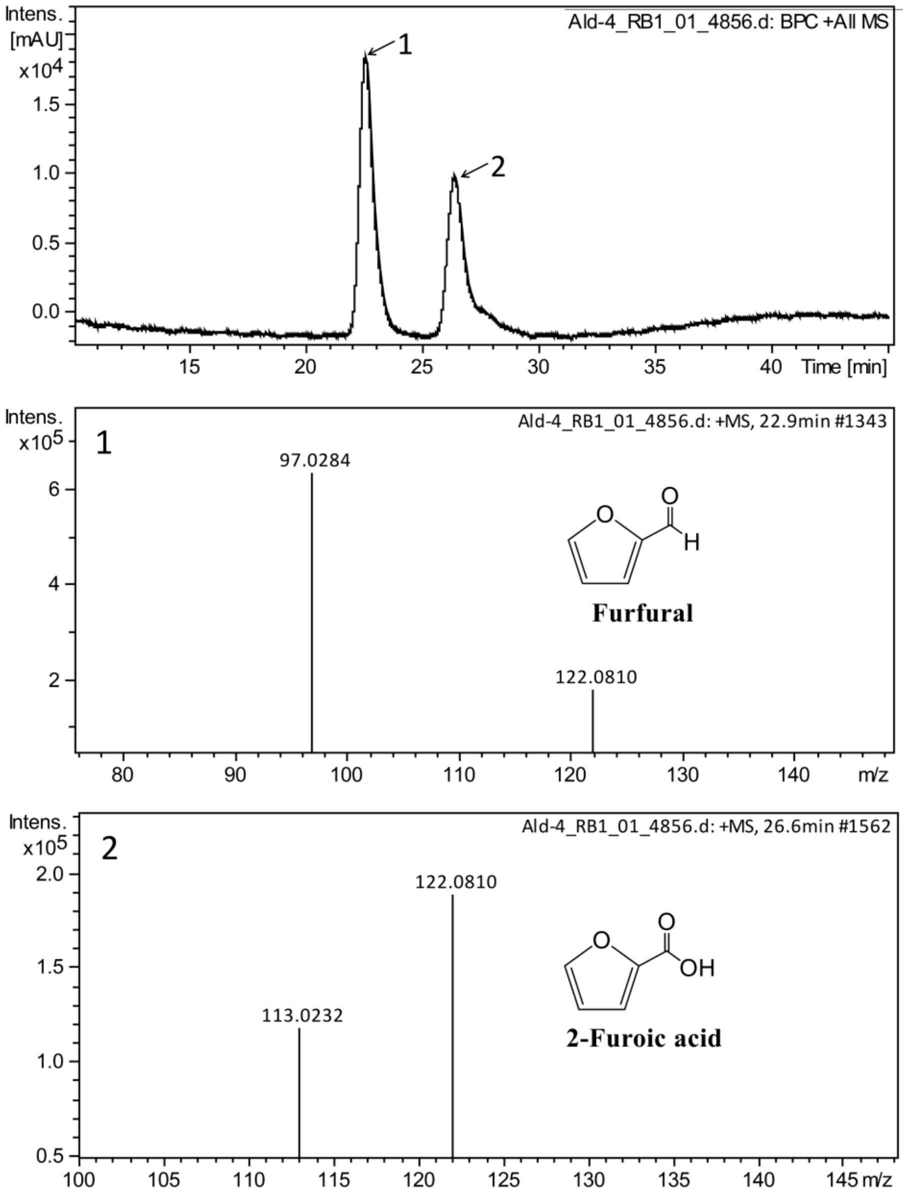

FIG S8 LC-MS profiles of Ald-catalyzed reaction with furfural as a substrate. The reaction mixture contained 50 mM Gly-NaOH buffer (pH 9.0), 3 mM furfural, 1.5 mM NAD^+, and 1.2 μM Ald. (1) Mass spectrum of the excess substrate furfural (*m/z* 97.0284). (2) Mass spectrum of 2-furoic acid (*m/z* 113.0232), which was the product of furfural dehydrogenation. Other peak (*m/z* 122.0810) is from the impurity.

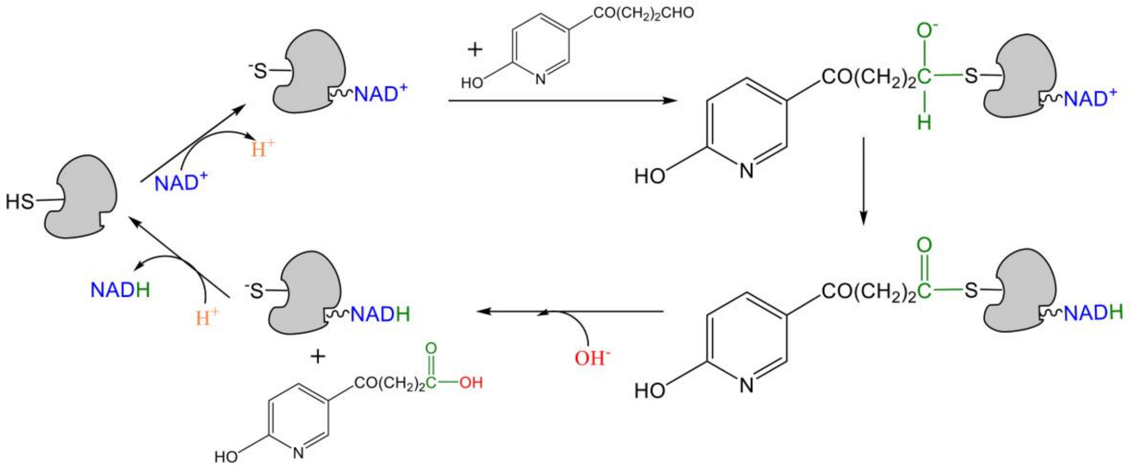

FIG S9 Proposed catalytic mechanism of Ald from *A. tumefaciens* S33.

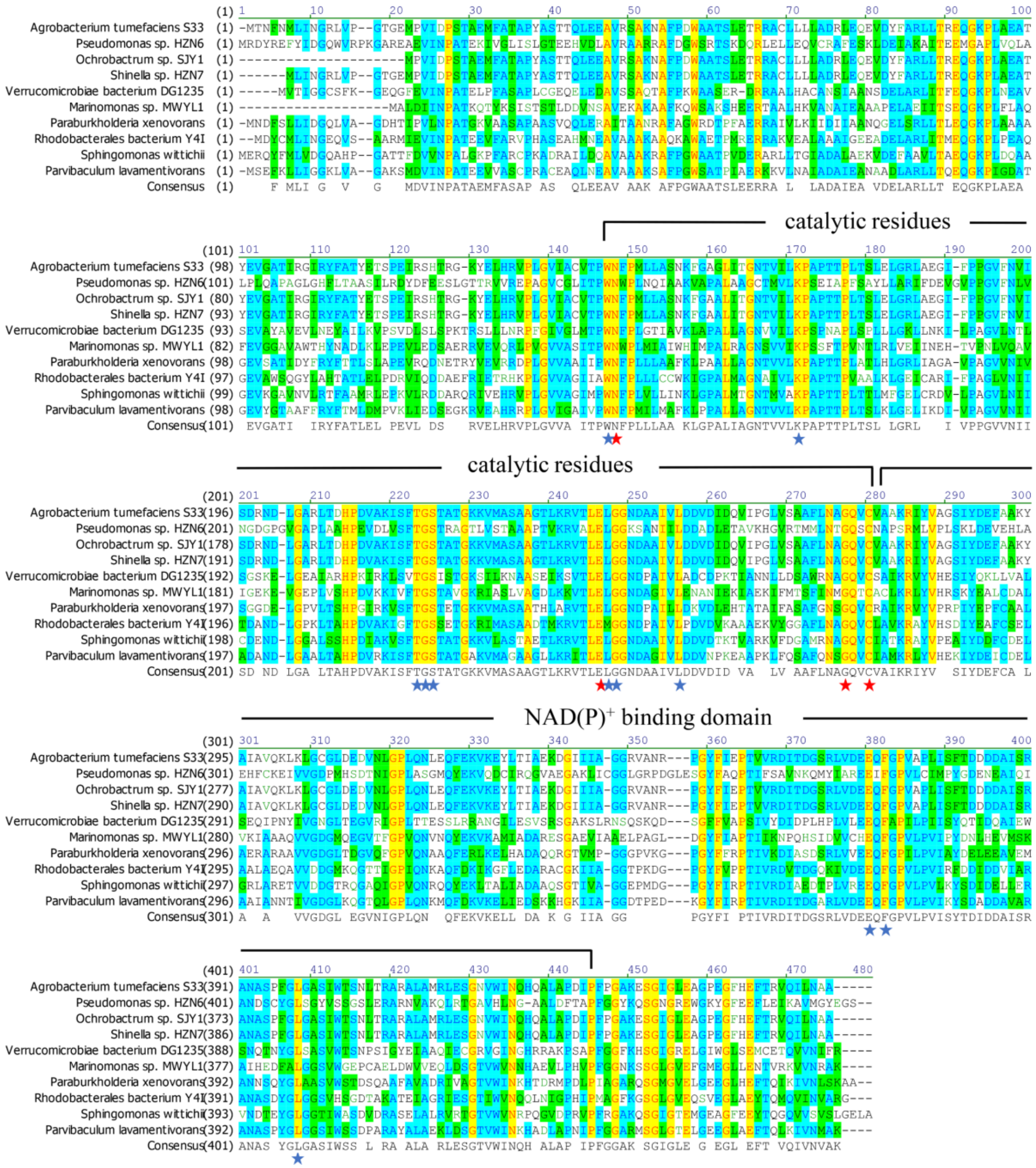

FIG S10 Protein sequences alignment of Ald proteins from different organisms. GenBank accession numbers: *Agrobacterium tumefaciens* S33 (AMD56947.1), *Pseudomonas sp.* HZN6 (AFD54464.1), *Ochrobactrum sp.* SJY1 (AIH15803.1), *Shinella sp.* HZN7 (AGS16699.2), *Verrucomicrobiae bacterium* DG1235 (EDY80708.1), *Marinomonas* sp. MWYL1 (WP_012070865.1), *Parvibaculum lavamentivorans* (WP_011995006.1), *Rhodobacterales bacterium* Y4I (EDZ44744.1),

Sphingomonas wittichii (WP_011952902.1), and *Paraburkholderia xenovorans* (WP_011493919.1). Sequences were obtained from NCBI (https://www.ncbi.nlm.nih.gov/) with GenBank accession numbers. The alignment of the ten sequences were performed with Vector NTI 10. Red stars (★) represent catalytic sites, and blue stars (★) represent $NAD(P)^+$ binding sites.

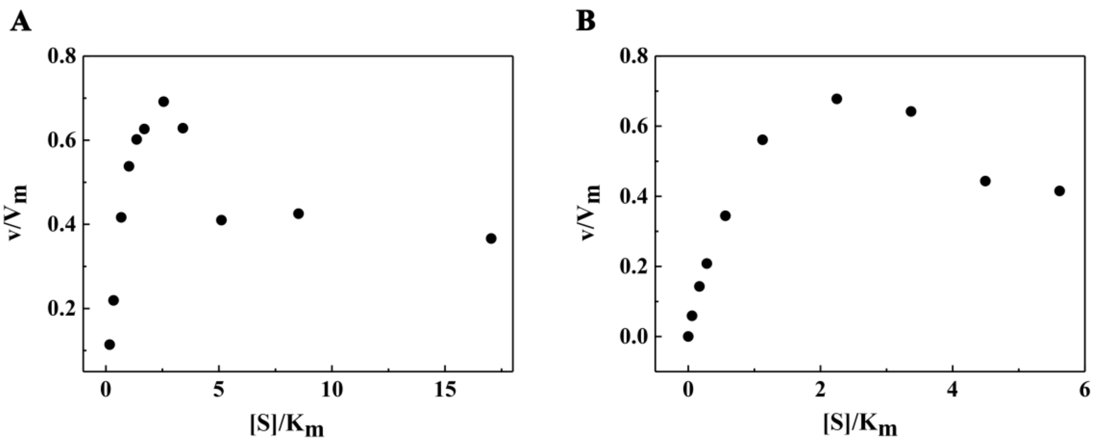

FIG S11 The substrate inhibition of Ald-catalyzed reactions with SAP (A) or benzaldehyde (B) as a substrate.
